# Gefitinib resistance resulted from STAT3-mediated Akt activation in lung cancer cells

**DOI:** 10.18632/oncotarget.1431

**Published:** 2013-11-24

**Authors:** Kai Wu, Qingshan Chang, Yongju Lu, Ping Qiu, Bailing Chen, Chitra Thakur, Jiaying Sun, Lingzhi Li, Anjaneyulu Kowluru, Fei Chen

**Affiliations:** ^1^ Department of Pharmaceutical Sciences, Eugene Applebaum College of Pharmacy and Health Sciences, Wayne State University, Detroit, MI, USA

**Keywords:** Gefitinib, EGFR, STAT3, Akt recovery

## Abstract

Hyperactivation of Epidermal Growth Factor Receptor (EGFR) tyrosine kinase is prevalent in human lung cancer and its inhibition by the tyrosine kinase inhibitors (TKIs), including gefitinib and erlotinib, initially controls tumor growth. However, most patients ultimately relapse due to the development of drug resistance. In this study, we have discovered a STAT3-dependent Akt activation that impairs the efficacy of gefitinib. Mechanistically, gefitinib increased association of EGFR with STAT3, which de-repressed STAT3 from SOCS3, an upstream suppressor of STAT3. Such a de-repression of STAT3 in turn fostered Akt activation. Genetic or pharmacological inhibition of STAT3 abrogated Akt activation and combined gefitinib with STAT3 inhibition synergistically reduced the growth of the tumor cells. Taken together, this study suggests that activation of STAT3 is an intrinsic mechanism of drug resistance in response to EGFR TKIs. Combinational targeting on both EGFR and STAT3 may enhance the efficacy of gefitinib or other EGFR TKIs in lung cancer.

## INTRODUCTION

Lung cancer is the leading cause of cancer-related mortality worldwide [1]. Lung cancer can be histologically classified into small cell lung cancer (SCLC) and non-small cell lung cancer (NSCLC), and the latter subtype constitutes 80% of lung cancers. Among all NSCLC patients, about 25% are estimated to harbor “activating mutations” in sequences encoding the epidermal growth factor receptor (EGFR) that causes a constitutive activation of the EGFR signaling pathway and thus provides tumor cells with an increased abnormal growth advantage. In order to target this abnormal hyperactivation, selective agents such as EGFR tyrosine kinase inhibitors (TKIs) had been developed, some of which, including gefitinib and erlotinib, had been approved by FDA for treatment of NSCLC [1]. This type of drugs selectively bind to the ATP binding pocket of the phosphorylation sites on the EGFR tyrosine kinase domain, thus suppress EGFR activation and block downstream signaling pathways. Gefitinib has shown measurable efficacy at early stage of treatment, but patients become insensitive to this drug after 6 to 9 months, which finally leads to treatment failure [2]. Several resistance mechanisms, such as EGFR T790M secondary mutation that leads to a higher ATP binding capacity, and MET amplification resulting in an aberrantly activated alternative pathway that bypasses the inhibited EGF receptors, had been discovered. Such resistance mechanisms occur in about 50% and 30% of resistant cases, respectively [3, 4]. However, the resistance mechanisms remain to be elucidated in at least 30% of all resistant cases. In addition, 2nd generation EGFR TKI (afatinib) aiming to overcome the EGFR T790M mutation has failed to show the expected clinical efficacy [5, 6]. Taken together, these previous studies implicate some other underlying mechanisms, such as nononcogenic or oncogenic dependent drug resistance [7, 8], existing alone or simultaneously with currently identified alterations of the EGFR signaling, may play an important role in the development of EGFR TKI resistance.

Signal transducer and activator of transcription-3 (STAT3) belongs to a protein family of transcription factor which are essential for cellular functions. Activation of STAT3 is determined by phosphorylation at tyrosine 705 residue and strengthened by phosphorylation at serine 727 residue [9-11]. Classically, two categories of pathways are mediating STAT3 tyrosine phosphorylation, one is receptor tyrosine kinase signaling, including EGFR, the other one is cytokine-signaling pathway, including IL-6/Janus-activated kinases (JAK) [9]. Accumulating evidence has demonstrated that aberrant expression and activity of STAT3 is implicated in both carcinogenesis and development of drug resistance in several cancer types, including NSCLC [10-15], suggesting that STAT3 may contribute to resistance to EGFR TKI treatment in lung cancer.

In this study, we have demonstrated that in human lung cancer cells, gefitinib treatment induces, rather than suppresses STAT3 activation as extrapolated from traditional EGFR signaling orthodox. We have further demonstrated that gefitinib not only promotes the direct interaction between EGFR and STAT3, which is needed for STAT3 activation, but also affects the upstream regulators of STAT3 in a dose-dependent manner. Low dose of gefitinib suppresses SOCS3 only while high dose inhibits both SOCS1 and SOCS3. As a result, activated STAT3 restores activation of Akt that was initially inhibited by gefitinib. Akt is an oncogenic protein kinase that is largely associated with cell survival and proliferation [16, 17]. Restoration of Akt activation eventually reduces sensitivity of the lung cancer cells to EGFR interruption. Extensive cell proliferation studies show that simultaneous inhibition of STAT3 sensitizes the cancer cells to gefitinib-induced repression of cell growth. Taken together, our data have suggested that gefitinib-induced STAT3 activation and subsequent Akt recovery may act as a novel mechanism of primary and possibly acquired resistance against gefitinib in NSCLC. Accordingly, combinational targeting of STAT3, Akt and EGFR may prevent or reverse drug resistance of EGFR TKI-based therapy in the lung cancers.

## RESULTS

### Gefitinib inhibited Akt initially followed by a recovery of Akt activation at the later time points

**Figure 1:**
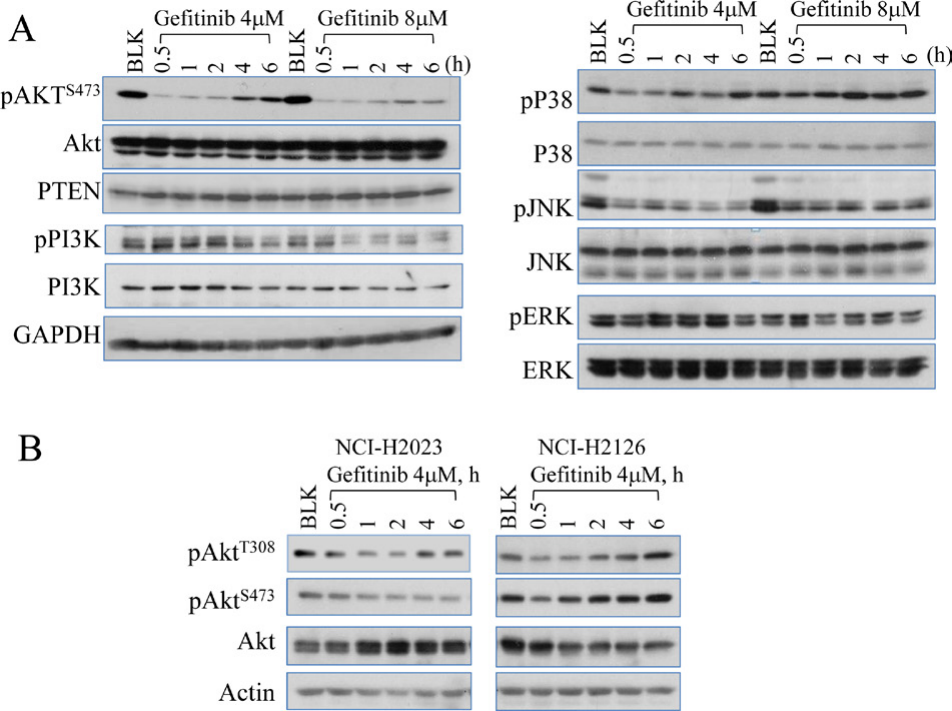
Gefitinib treatment on Akt inhibition and recovery (A) Westernblotting analysis shows that gefitinib inhibits multiple protein regulators involved in EGFR signaling pathway and induces Akt recovery in A549 cells (human lung cancer cell line). The cells were either untreated (blank, BLK) or treated with 4 or 8 µM gefitinib for 0.5 to 6 h. The recovery at the later time points of gefitinib treatment can be seen only for Akt, but not PI3K, p38, JNK, and ERK. (B) Westernbloting data showed Akt recovery in NCI-H2023 cells and NCI-H2126 cells treated with gefitinib for the indicated hours (h).

Activation of EGFR has been linked to the pro-survival signaling pathways, including Akt, STAT3 and RAF/MEK/ERK mitogen-activated protein kinase (MAPK) [16-18]. To monitor the effect of EGFR inhibition on these downstream pathways, we performed time course studies in the A549 cells treated with gefitinib, a selective inhibitor of the tyrosine kinase site in the catalytic domain of EGFR. The Akt activation was inhibited by gefitinib at the earlier time points (0.5 to 2 h). However, the inhibited Akt activation was gradually recovered at the later time points (4 to 6 h) (Fig. 1A). In contrast, gefitinib showed a sustained inhibition on the upstream kinases of Akt, including PI3K and c-Jun N-terminal kinase (JNK), without notable recovery in the later time points (Fig. 1A), which is consistent with previous reports [17, 19]. Gefitinib treatment shows relatively limited effects on the activity of ERK and p38 mitogen-activated protein kinase (p38), even when the cells were treated with a higher concentration of gefitinib of up to 8 μM. These data suggest that the recovery of Akt activation at the later time points of gefitinib treatment is not a result of the recovered activation of the upstream kinases, such as PI3K and JNK.

**Figure 2:**
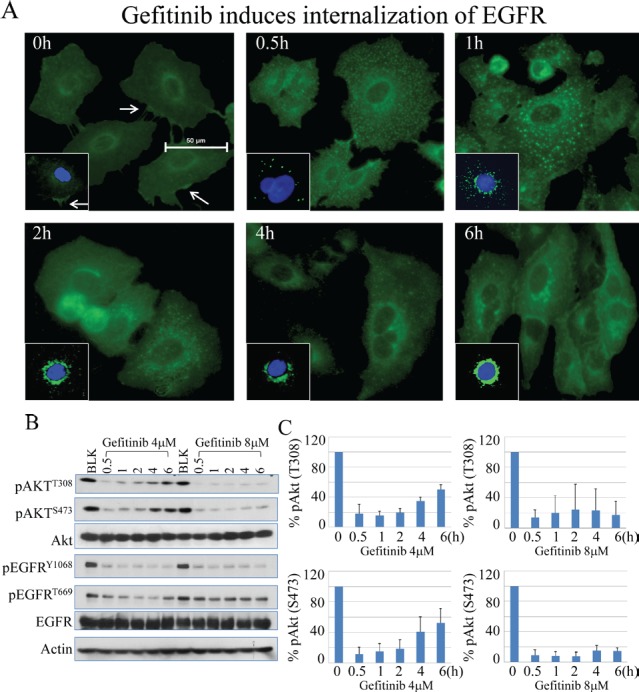
Gefitinib inhibits EGFR constitutively and substantially (A) Immunofluorescence test shows the process of internalization of EGFR at proceeding time points after treatment of gefitinib in A549 cells. (B) Gefitinib treatment induced continuous inhibition of EGFR phosphorylation on tyrosine 1068 (Y1068) and threonine 669 (T669) without recovery at the later time points. (C) Semi-quantification of Akt recovery following gefitinib treatment.

To determine whether this Akt recovery following gefitinib treatment is a common phenomenon among the lung cancer cell lines, we also evaluated Akt activation in two additional NSCLC cell lines, NCI-H2023 and NCI-H2126, both of which are derived from adenocarcinoma lung cancer. As indicated in Fig. 1B, a similar later time Akt recovery as what had been noted in A549 cells was observed in both NCI-H2023 and NCI-H2126 cells treated with gefitinib. However, a subtle difference in Akt recovery were measured among these different lung cancer cell lines, e.g., while both NCI-H2126 and A549 showed a clear later time recovery of Akt phosphorylation at threonine (T) 308 (pAkt^T308^) and serine (S) 473 (pAkt^S473^) (Figs. 1B and 2B), NCI-H2023 exhibited recovery of pAkt^T308^ only following gefitinib treatment. No recovery of pAkt^S473^ was noted in NCI-H2023 cells. This difference possibly resulted from different gene mutation status among these cell lines. Although all of these cells express high level of the wild-type EGFR, both A549 cells and NCI-H2126 cells also harbored active mutations of Kras oncogene [20, 21].

#### Akt recovery is not due to re-activation of the EGFR by gefitinib

EGFR has been viewed as one of the key upstream kinases responsible for growth factor-induced Akt activation [22]. To determine whether the recovery of Akt activation is due to failed inhibition of EGFR by gefitinib at the later time points, we measured the levels of internalization and phosphorylation of EGFR in response to gefitinib. In immunofluorescent staining assay, gefitinib treatment induced a fast and sustained internalization of the EGFR (Fig. 2A). Treatment of the cells with 4 μM gefitinib, a gradual translocation of the EGFR from cell membrane to intracellular vesicles and finally randomly distributed at the perinuclear area was observed, indicating a constitutive and effective inhibition of the EGFR by gefitinib.

To further validate the inhibitory effect of gefitinib on EGFR, we next measured the phosphorylation status of the EGFR in the cells treated with gefitinib. Again, the time course studies had demonstrated a rapid recovery of Akt phosphorylation in both serine 473 (S473) and threonine 308 (T308) residues within 6 h following the initial inhibition, esp. in the cells treated with 4 μM gefitinib (Fig. 2B). Semi-quantification of the Akt phosphorylation suggested about 40-60% recovery of Akt activation at the 4 to 6 h time points of gefitinib treatment (Fig. 2C). However, there is no similar recovery pattern of EGFR phosphorylation following gefitinib treatment. At both 4 and 8 μM gefitinib treatments, phosphorylation of Y1068 and T669 of EGFR was substantially inhibited from the earlier to later time points (Fig. 2B). These data, thus, suggest that the Akt recovery is not a result of failed inhibition of EGFR by gefitinib either.

#### Gefitinib induces STAT3 activation in lung cancer cells

**Figure 3:**
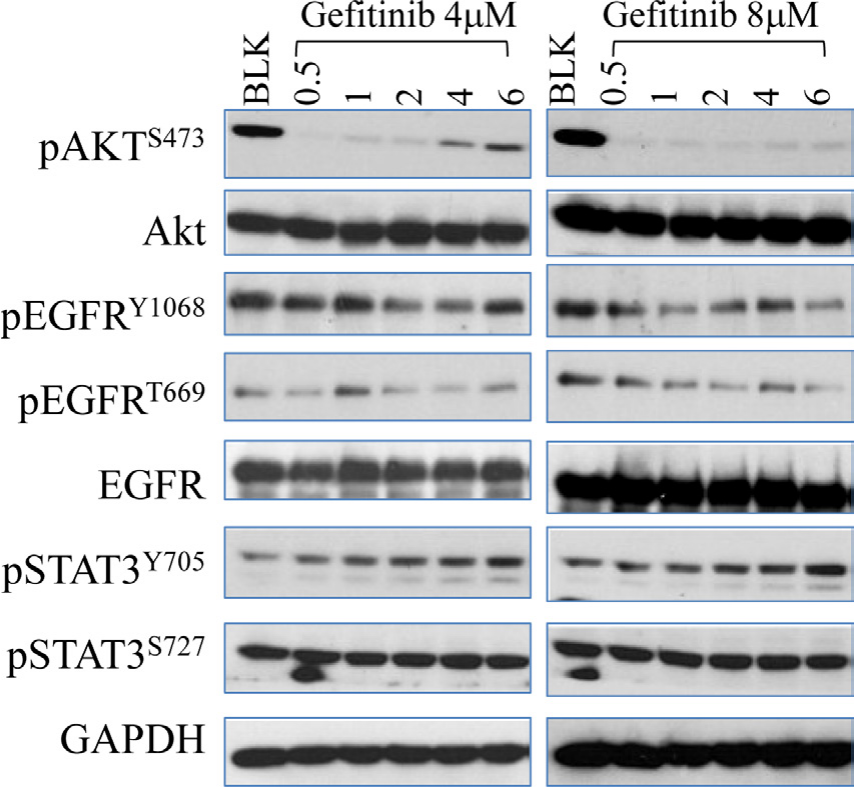
Gefitinib inhibits EGFR phosphorylation but induces STAT3 activation in lung caner cells Cells were subject to time-dependent treatment of gefitinib at 4μM (left panel) and 8μM (right panel) for the indicated time periods. The expression and activity of EGFR, Akt and STAT3 in A549 cells were determined by Western Blot.

STAT family proteins, STAT3 in particular, play an essential role in EGFR-mediated cellular responses [16]. Emerging evidence has demonstrated that hyperactivation of STAT3 contributes to carcinogenesis in a variety of cancer types and disruption of the STAT3 signaling decreased tumor growth [23, 24]. Inhibition of EGFR by gefitinib is supposed to be able to down regulate the subsequent STAT3 activity. However, time course test showed that gefitinib treatment, in fact, induces STAT3 activation in A549 cells (Fig. 3). After exposure to gefitinib at both 4μM and 8μM, a rapid increase of phosphorylation of STAT3 in tyrosine 705 residue (Y705) was observed and the intensity of which is not affected by different doses of gefitinib. Interestingly, the trend of gefitinib-induced STAT3 activation is accordant with the recovery pattern of Akt after gefitinib treatment, indicating potential interaction between these two pathways (Fig.3)

#### Inhibition of STAT3 prevented recovery of Akt activation in gefitinib-treated cells

**Figure 4:**
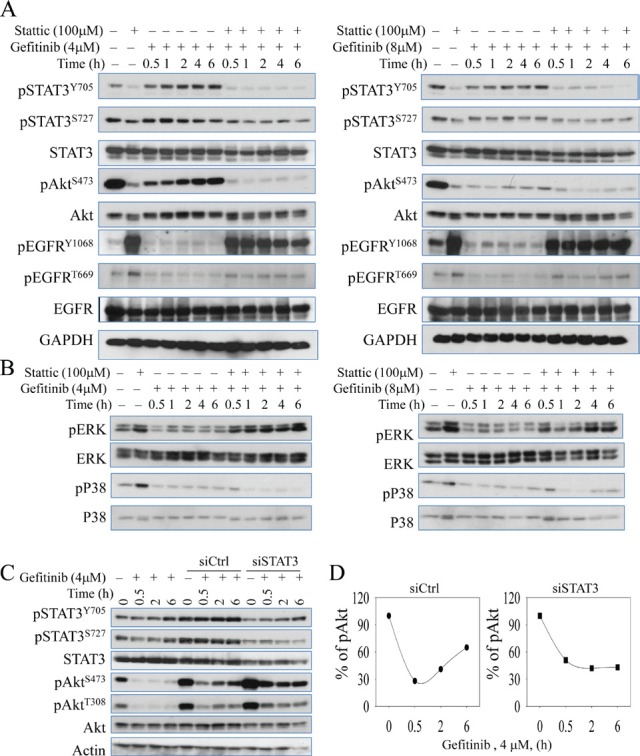
Chemical inhibitor and gene silencing of STAT3 suppresses succeeding recovery of Akt activation after gefitinib treatment (A & B) Immunoblotting analysis of expressions and activities of EGFR, Akt, STAT3, ERK, and P38 under time-dependent treatment with 4μM (left panel) and 8μM (right panel) gefitinib combined with or without 100 μM Stattic (STAT3 inhibitor) for up to 6 hours in A549 cells. (C) Silencing STAT3 by siRNA diminishes gefitinib-induced Akt recovery in A549 cells. (D) Semi-quantification of the Akt S473 phosphorylation in the cells treated with gefitinib and transfected with control siRNA (siCtrl, left panel) or STAT3 siRNA (siSTAT3, right panel).

It has been well-documented that STAT3 signaling pathway contributes to Akt activation in response to a number of extracellular and intracellular signals [25]. More recently, STAT3-Akt activation loop has been uncovered in lung epithelial cells [26-28]. Based on that rationale, we hypothesize that gefitinib-induced STAT3 activation is responsible for the sequential recovery of Akt phosphorylation. To test that, we co-treated cells with Sttatic, a STAT3 inhibitor, which potently down regulates its phosphorylation without affecting the total amount of STAT3. As shown in the Figs.4A and 4B, when STAT3 function is inhibited, the recovery pattern of Akt is also eliminated even when EGFR is hyperactivated possibly by the treatment of STAT3 inhibitor, Sttatic. In addition, Sttatic co-treatment does not exhibit significant inhibitory effects on ERK and P38 phosphorylation at the same time (Fig. 4B), suggesting that Akt recovery is specifically dependent on STAT3 function. In order to exclude the potential off-target effects of the chemical inhibitor, we further employed a siRNA-based gene silencing strategy to confirm the above observation. When the cells were transfected with STAT3 specific siRNA, siSTAT3, the total amount and activity of STAT3 are reduced, and the recovery pattern of Akt is eliminated, although the basal level of Akt phosphorylation is elevated. In contrast, the cells transfected with control siRNA or without transfection show no inhibitory effects on either STAT3 activation or the Akt recovery (Figs.4C & 4D).

#### Gefitinib promotes physical binding of STAT3 to EGFR

**Figure 5:**
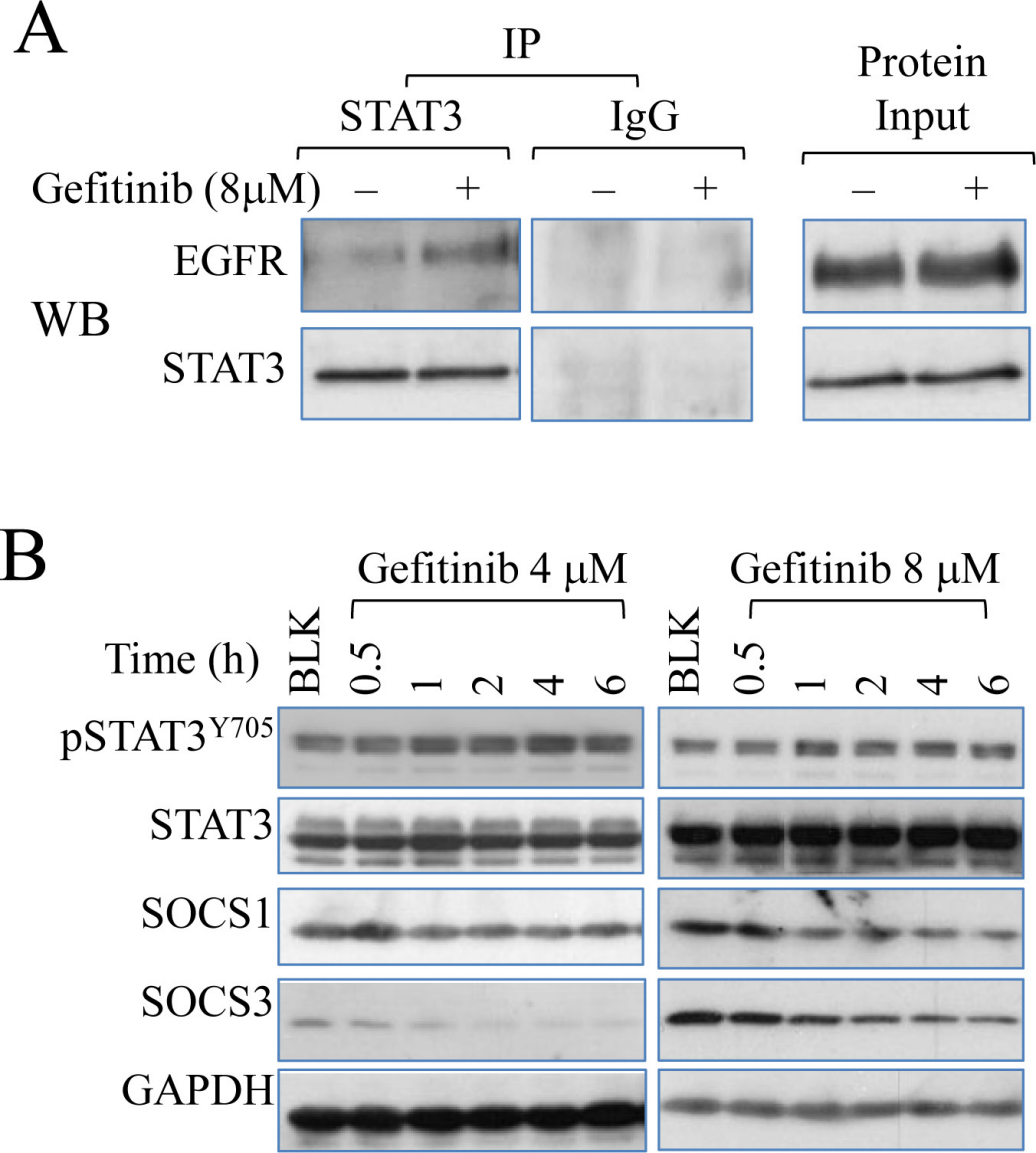
Gefitinib promotes EGFR-STAT3 interaction (A) Immunoprecipitation assay (left pannel) demonstrates direct physical binding of EGFR and STAT3 induced by gefitinib treatment. Cells were treated with 8μM gefitinib for 6 hours. The samples were precipitated with STAT3 antibody and detected with antibodies against EGFR and STAT3 sequentially. (B) Immunoblotting analysis shows the effect of gefitinib on SOCS1 and SOCS3 in A549 cells.

Prompted by the activation of STAT3 by gefitinib, we then sought to identify the mechanism of how gefinitib activates STAT3. In receptor tyrosine kinase-dependent signaling, STAT3 activation is increased by binding to certain STAT3 docking sites on EGFR c-terminal domains [29]. To determine the direct physical interaction between STAT3 and EGFR, immunoprecipitation assay was performed. As shown in Fig.5A, gefitinib treatment induced a stronger binding of STAT3 to EGFR when identical amount of total protein is used for pull down by anti-STAT3 antibody. Another fundamental signaling pathway leading to STAT3 activation is cytokine pathway in which STAT3 is activated by Janus Kinase (JAK) family proteins, which is negatively regulated by the suppressor of cytokine signaling proteins (SOCS), such as SOCS1 and SOCS3. In order to identify the potential role of the regulators in cytokine-activated pathway, we carried out another time course study to determine the levels of the SOCS proteins. Level of SOCS3 is reduced in cells treated with 4μM and 8μM gefitinib, while significant reduction of SOCS1 is observed in 8μM group only (Fig. 5B), suggesting that gefitinib is able to inhibit SOCS proteins in a manner of dose-dependency, which accounted for an alternative mechanism contributing to gefitinib-induced STAT3 activation.

#### STAT3 inhibition sensitizes lung cancer cells to gefitinib treatment in vitro

**Figure 6:**
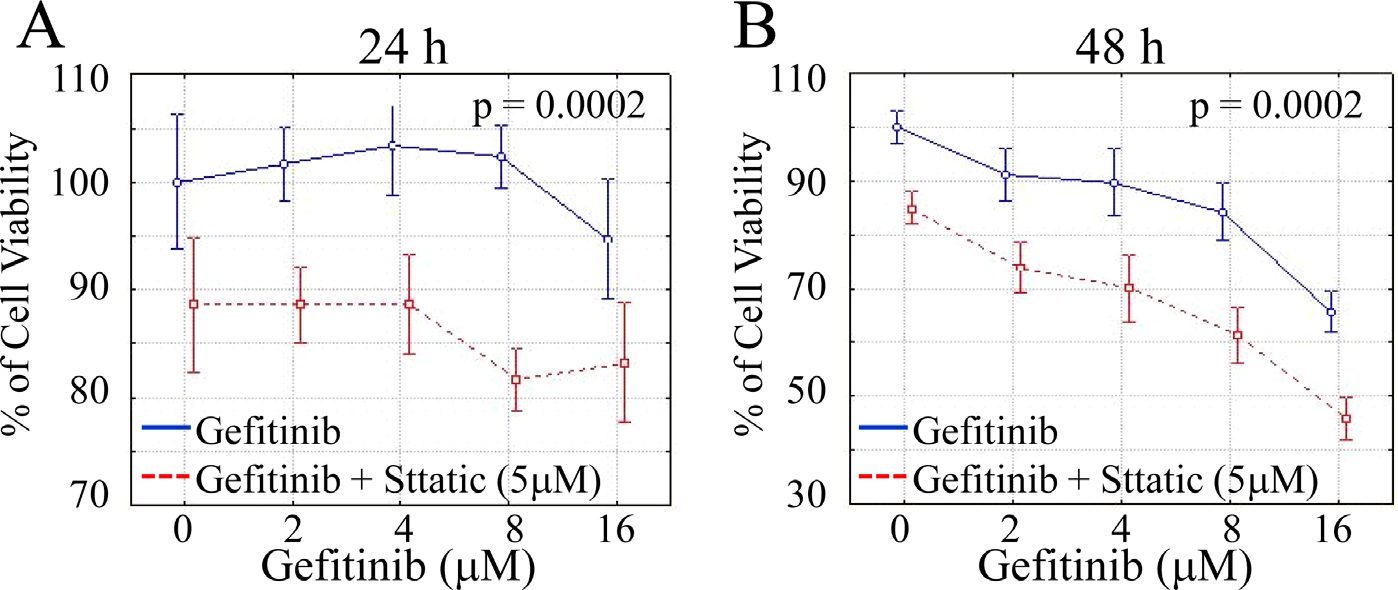
STAT3 inhibitor enhances the inhibitory effect of gefitinib on cell growth CyQUANT NF Cell Viability Assay Kit (Invitrogen) was used to stain viable cells. Data show the relative percentage of viable A549 cells after exposed to gefitinib ranging from 2μM to 16μM in the absence or presence of Stattic for 24 hours (A) and 48 hours (B), respectively. (P<0.01 in both tests)

Since gefitinib has been shown to induce STAT3 activation and subsequent Akt recovery (Fig.2 and Fig.3), we went to assess the anti-tumor efficacy of combinational inhibition of EGFR and STAT3. A549 cells were exposed to dose-dependent treatment of gefitinib (2-16μM) in combination with STAT3 inhibitor (5μM) for 24 h and 48 h, respectively, before cell viability was examined and analyzed. As shown in Fig.6, combinational STAT3 inhibition significantly fortifies the anti-cell growth effects of gefitinib in lung cancer cells compared to the group of gefitinib alone (P<0.01 in both tests). These results indicate that combinational targeting of STAT3 may be an effective method to overcome the intrinsic insensitivity to EGFR TKI therapy of lung cancer cells.

### DISCUSSION

Drug resistance remains a major obstacle to successful cure of NSCLC via EGFR TKI-based therapies since it occurs in almost all NSCLC patients including those initially sensitive to EGFR targeting therapy after 6-9 months treatment. The subsequent relapse and progression of disease finally leads to treatment failure. The fact that abnormalities leading to resistance to gefitinib frequently overlap and that efforts aiming to overcome defined resistance mechanisms show limited efficacy both *in vitro* and *in vivo*, indicating possibilities of other mechanisms which are crucial for occurrence and maintenance of resistance against EGFR TKI in NSCLC [5]. Explorations into such mechanisms are of scientific and clinical significance.

It has long been believed that EGFR TKIs, such as gefitinib, function through selectively binding to the tyrosine kinase domain on EGFR and suppressing its major downstream pro-survival and anti-apoptosis signaling pathways, including STAT3, Akt and ERK [16, 17, 19]. In this study, however, we highlight the gefitinib-induced STAT3 activation in NSCLC cell lines A549, NCI-H2023 and NCI-H2126, which is against the classic knowledge of tyrosine kinase-dependent pathway of STAT3 activation [16]. In addition, based on previously defined STAT3-Akt axis in lung epithelial cells [26], we have further demonstrated that phosphorylation level of Akt substantially recovered rapidly from initial inhibition within 6 hours after gefitinib treatment and this process is dependent on the synchronous gefitinib-induced STAT3 activation. Considering the pivotal role of STAT3 and Akt in anti-apoptotic machinery [23, 25, 30, 31], our study answers, at least partly, why certain types of lung cancer cells are resistant to gefitinib-induced cell death. Moreover, this notion has been further substantiated by the cell viability assay using combinational inhibition of EGFR and STAT3. Even slight inhibition (5μM) of the latter can significantly enhance the anti-tumor efficacy of gefitinib, especially at low doses, indicating a promising synergistic strategy to control resistance to gefitinib. On the other hand, this study also sheds light on the acquired resistance of gefitinib. It is likely that lung cancer cells take advantage of or even strengthen this gefitinib-induced STAT3-Akt axis through long term exposure to gefitinib in order to gain resistance to it. This hypothesis is partly confirmed by the observation that STAT3 is hyperactivated in tyrosine 705 residue in gefitinib-resistant lung cancer cell line derived from wild type A549 cells [32]. Increased STAT3 phosphorylation has also been observed in EGFR mAb treatment-resistant cell models of head and neck squamous carcinoma (HNSCC) and bladder cancer [33]. Furthermore, gefitinib-induced STAT3-Akt axis defined in this study also provides explanation for the mechanism of dramatic efficacy of combinational suppression of STAT3 or subsequent Akt/mTOR in overcoming acquired resistance in both *in vitro* and *in vivo* lung cancer models receiving EGFR TKI-based therapy [34-36].

Activation of STAT3 is achieved from both receptor tyrosine kinase pathway, including EGFR-centered signaling, and cytokine signaling pathway, for instance, Interleukin-6/JAK/STAT3 pathway [9]. In an effort to explore the mechanisms underlying gefitinib-induced STAT3 activation, we demonstrate that gefitinib not only promotes the direct binding of EGFR and STAT3 but also, surprisingly, affects the receptor tyrosine kinase-independent pathway of STAT3 activation. Multiple tyrosine residues on the cytoplasmic region of EGFR, including Y1068, Y1086 and Y1045, have been identified as docking sites where STAT3 uses its SH2 and DNA-binding domains to interact with EGFR and gets activated [29]. In agreement with such notion, our study shows that gefitinib treatment is able to directly promote the physical interaction between EGFR and STAT3, and thus regulates STAT3 activity in A549 cells. More interestingly, we have also revealed that gefitinib down regulates another important upstream regulator of STAT3, the SOCS family proteins. As shown in Fig. 5, gefitinib at 4μM is able to reduce the level of SOCS3, while higher concentration (8μM) is required to more effectively suppress both SOCS1 and SOCS3, suggesting that gefitinib also induces STAT3 activation by altering cytokine signaling of its activation. Considering SOCS proteins are also recruited by certain regulatory region of EGFR, extending from Y1114 to E1172, to block STAT3 activation [29], reduced SOCS proteins by gefitinib may also abrogate the intrinsic inhibitory effects of EGFR on STAT3.

The STAT3 activation and the subsequent Akt recovery may be one of the key mechanisms of therapy-induced tumor progression in the lung cancer patients who received EGFR TKI treatment. Both STAT3 and Akt are important protein kinases contributing to either oncogenic or nononcogenic chemodrug resistance that fosters generation of the cancer stem cells or selection of the fast growing cancer cells [7, 8]. At the present, there is limited evidence showing that gefitinib resistance is resulted from reprogramming of the cancer cells to form cancer stem cells that not only replenishes the tumor mass but also causes clonal shifts of the cancer cells from drug sensitive cells to drug resistant cells. Thus, future studies are much needed to determine whether the gefitinib resistant lung cancer cells have the features of the cancer stem cells. Considering the facts that both STAT3 and Akt are critical kinases for the self-renewal and pluripotency of the cancer stem cells [37, 38], it is plausible to combine gefitinib with agents that target STAT3 and Akt to prevent gefitinib resistance and the faster relapse of the tumors.

In NSCLC, differences in mutation status of EGFR, including “activating mutations” and secondary mutations, and preference in dependence of EGFR signaling, are fundamental factors determining sensitivity to gefitinib [19, 39-41]. Established evidence has suggested an amplified expression of the wild-type EGFR is more frequent in prevalence yet associated with less sensitivity to gefitinib treatment. The results of this study have revealed a new mechanism of resistance to gefitinib, especially in cells with an overexpressed wild-type EGFR. In the future, the role of gefitinib-induced STAT3-Akt activation loop needs to be further tested among the NSCLC cells with different EGFR statuses, which will provide deeper insights into our knowledge of drug resistance in NSCLC and provide valuable information to optimize anti-tumor therapy in lung cancer patients.

## MATERIAL AND METHODS

### Cell culture and reagents

The human lung carcinoma cell line A549, NCI-H2023, NCI-H2126, and bronchial epithelial cell line BEAS-2B were purchased from the American Type Culture Collection (ATCC) (Manassas, VA) and were cultured in F12K medium or DMEM medium (ATCC, Manassas, VA) supplemented with 10% fetal bovine serum (Invitrogen, Grand Island, NY) and 1% penicillin-streptomycin (Sigma, St. Louis, MO). Cells were maintained in humidified incubator at 37°C with 5% CO_2_. STAT3 inhibitor V (Sttatic) was purchased from Santa Cruz Biotechnology, Inc. (Santa Cruz, CA).

### siRNA transfection

Total of 4×10^5^ cells per well were seeded into 6-well plates and incubated until they reached 50% confluency. siRNAs at a final concentration of 100nM were then forward-transfected using Lipofectamine RNAiMAX (Invitrogen) following manufacturer protocol. Cells were cultured for 24 hours for gene silencing followed by sequential treatment of gefitinib. siRNA against STAT3 and control siRNA were purchased from Cell Signaling (Danvers, MA).

### Western Blotting

Cells were lysed by 1×RIPA cell lysis buffer (Cell Signaling) supplemented with protease and phosphatase inhibitors cocktail (Roche, Indianapolis, IN) and 1mM PMSF. Collected cell lysates were then homogenized by sonification and insoluble debris was removed through centrifugation of 13,000g at 4 °C for 15 minutes. The concentrations of protein were then determined using Pierce BCA Protein Assay Kit (Thermo Scientific, Rockford, IL). The protein samples were prepared using 4 × LDS sample buffer (Invitrogen) with dithiothreitol at a final concentration of 200 mM and were denatured by boiling at 95°C for 5 minutes before separation by 7.5%, 10% or 12% SDS-PAGE gel, where appropriate. Separated samples were then transferred onto PVDF membrane (Invitrogen) and blocked with 5% nonfat milk diluted in TBST for 1 hour at room temperature. After extensive washing with TBST, the membranes were incubated with indicated primary antibodies for overnight at 4°C and corresponding alkaline phosphatase (AP)-coupled second antibodies for 1 hour at room temperature before detecting. CDP-Star Reagent (New England Biolabs) was used to visualize the signals on autoradiography films. Primary antibodies against phospho-Akt (Ser473), phospho-Akt (Thr308), total Akt, phospho-STAT3 (Ser727), phospho-STAT3 (Tyr705), total STAT3, phospho-EGFR (Tyr1068), phospho-EGFR (Thr669), total EGFR, phospho-PI3K (Tyr458), PI3K, PTEN, phospho-p38 (Thr180/tyr182), p38, phospho-Erk (1/2) (Thr202/tyr204), Erk, phospho-JNK (Thr183/Tyr185), JNK, GAPDH, β-actin, and AP-linked mouse IgG were purchased from Cell Signaling (Danvers, MA). Antibodies against SOCS1 and SOCS3 were purchased from Millipore (Temecula, CA) and abcam (Cambridge, MA), respectively.

### Immunofluorescent staining

Fifty thousand A549 cells per well were plated into 24-well plates. Cells were allowed to grow and attach for 24 hours before time-dependent treatment with 4μM gefitinib for up to 6 hours and sequential fixation with 4% formaldehyde for 15 min at room temperature. After brief washing with PBS, cells were blocked in 1×PBS containing 5% normal goat serum and 0.3% Triton X-100 for 1 hour and EGFR antibody for an additional 1 hour. Then cells were incubated in Alexa Fluor 488 or FITC-linked goat anti-rabbit IgG (Invitrogen) for 1 hour in dark. One drop of Prolong Gold antifade reagent with DAPI (Invitrogen) was added to each well before photography.

### Immunoprecipitation

Cells were lysed in non-denaturing lysis buffer containing 137 mM NaCl, 20 mM Tris-HCl (pH8.0), 10% glycerol, 2 mM EDTA and 1% NP-40 supplemented with protease and phosphatase inhibitors cocktail (Roche). After gentle agitation for 30 minutes and purification by centrifugation of 13,000g, the lysates were pre-cleared with rabbit IgG (Santa Cruz) and protein A/G plus beads (Santa Cruz). Eight hundred μg of protein for each sample was incubated with indicated antibodies at a dilution ratio of 1:100 at 4°C for overnight. The protein samples were further incubated with 40 μL of protein A/G plus beads (Santa Cruz) for 4 hours at 4°C, followed by 3 washes with non-denaturing lysis buffer. The prepared samples were then detected with Western Blotting as described above.

### Cell proliferation assay

5 × 10^3^ A549 cells diluted in 100 μL full growth medium were seeded into 96-well plate. After 24 hours, 100 μL medium containing 2× indicated concentration of gefitinib with or without STAT3 inhibitor was added to each well and each dose was added to 3 wells. CyQUANT NF Cell Proliferation Assay Kit (Invitrogen) was used to stain viable cells. After 30 minutes in dark, the intensity of fluorescence was measured using BioTek Synergy 2 plate reader (BioTek, Winooski, VT).

### Statistical analysis

Results of quantification of immunoblotting data was analyzed by Student's t-test and shown as mean ± SD. Cell proliferation data was processed using one-way ANOVA and the statistical significance of differences in inhibitory effects between different treatments and samples were determined by Post-hoc tests. And under both circumstances, p < 0.05 is considered as statistically significant.
